# Prediction of injuries, traumas and musculoskeletal pain in elite Olympic and Paralympic volleyball players

**DOI:** 10.1038/s41598-023-38112-x

**Published:** 2023-07-08

**Authors:** Anna Zwierzchowska, Eliza Gaweł, Miguel-Angel Gómez, Aleksandra Żebrowska

**Affiliations:** 1 Institute of Sport Sciences, The Jerzy Kukuczka Academy of Physical Education in Katowice, Katowice, Poland; 2 Facultad de Ciencias de la Actividad Física y del Deporte-Inef Madrid, Universidad Politécnica de Madrid, Madrid, Spain

**Keywords:** Musculoskeletal system, Risk factors, Public health

## Abstract

The study aimed to identify the prevalence and location of injuries, traumas, and musculoskeletal complaints in Paralympic and Olympic volleyball players with different impairments and initial playing positions (sitting/standing); and to identify the predictors of the abovementioned variables using a multivariate CRT model. Seventy-five elite volleyball players from seven countries took part in the study. They were divided into three study groups: (SG1)—lateral amputee Paralympic volleyball players, (SG2)—able-bodied Paralympic volleyball players, (SG3)—able-bodied Olympic volleyball players. The prevalence and location of the analyzed variables were assessed with surveys quessionaires, while game-related statistics was interpreted based on the CRT analysis. Regardless of the impairment or initial playing position, both the humeral and knee joints were found to be the most frequent locations of musculoskeletal pain and/or injuries in all studied groups, followed by LBP. Players from SG1 and SG3 were characterized by an almost identical prevalence of reported musculoskeletal pain and injuries, what was not noted in SG2. Extrinsic compensatory mechanism (playing position) may be a crucial variable for prediction of musculoskeletal pain and injuries in volleyball players. Lower limb amputation seems to impact the prevalence of musculoskeletal complaints. Training volume may predict the prevalence of LBP.

## Introduction

Volleyball is a dynamic team sport that requires the power of the upper and lower limbs and agility to perform specific technical tasks (mostly with the dominant upper and lower limbs) and multidirectional upright locomotion^1^. Although playing position in volleyball may require different actions performed by different players, vertical jumping performance is known as a key factor to succeed in Olympic volleyball as it is one of the components of movement when attacking, defending, and serving^2^. However, because of the necessity of performing multiple jumps and multidirectional locomotion during a traditional volleyball game, this action is inaccessible to people with disabilities, especially those with motor impairments of the lower limbs. Thus, modified rules of Paralympic volleyball include, among others, different initial player’s position, that is determined by the contact of the player’s buttocks with the floor on the court in the sitting position when touching a ball^3^. Furthermore, players only use their upper limbs for performing technical behaviors and changing positions on the court. Due to the modified rules of the game, sitting volleyball is considered a highly competitive team sport that has a faster pace than Olympic volleyball (due to e.g., lower net height)^4^.

Regardless of the type of rules (Olympic or Paralympic), volleyball is believed not to be a particularly dangerous physical activity compared to other team sports like handball or basketball due to not involve physical contact or rough play with the opponent during the game actions^5^. Nevertheless, the currently available scientific literature suggests that among Olympic volleyball players, the number of musculoskeletal complaints and injuries is constantly increasing^6,7^ and the injuries are located mostly in ankle, shoulder and knee joints^6,8^, in which both acute and overuse injuries occur. Moreover, scientific studies indicate that the total rate of musculoskeletal injuries in volleyball players rate from 1.7 to 10.7 injuries/1000 player hours^6^ whay may be related to both the contact with another player while landing in the conflict zone between the net and the foot of the opposite player^9–11^, and by the incidence of body’s compensatory mechanisms (internal and external)^12,13^. According to the meta-analysis by Zwierzchowska et al.^13^ and the studies of Grabara^14^ volleyball training can couse musculoskeletal adaptations to sport specific training (extrinsic compensatory mechanism), that thereafter can heighten the risk of musculoskeletal pain, injuries and traumas.

Nevetheless, for Paralympic volleyball, the data that addressed the abovementioned issues remains limited. Some research suggests that the overall number of musculoskeletal complaints, injuries, and traumas among elite Polish sitting volleyball players is high^12^, but at the same time it indicates the need for futher and deeper analyses. Even though the possible determinants of musculoskeletal pain and injuries have been examined, there is still a lack of studies to evaluate this issue in terms of initial playing position i.e. sitting versus standing and lower limb impairments. At the same time, finding a single study to analyze the predictors of the incidence of injuries, traumas, and musculoskeletal complaints in Paralympic volleyball athletes is difficult.

Based on the above rationale and the gap existing in the available scientific literature, it seems reasonable to focus attention on the importance of injuries, traumas, and musculoskeletal pain prediction in elite-level sports. Therefore, the study aimed to (1) identify the prevalence and location of injuries, traumas, and musculoskeletal complaints in Paralympic and Olympic volleyball players with different impairments and initial playing positions (sitting/standing); and (2) to identify the predictors of the abovementioned variables using a multivariate model of classification and regression tree (CRT). It was hypothesized that regardless of the impairment or initial playing position (sitting/standing), both injuries and musculoskeletal pain occur mostly in the humeral joint and lower back. At the same time, it was assumed that both lower limb impairment and initial playing position (sitting/standing) are crucial variables for the prediction of the incidence of injuries, traumas, and musculoskeletal pain.

## Materials and methods

### Participants

Seventy-five (female = 20; male = 54; age = 34.1 ± 11.1; body height = 1.83 ± 0.1; body mass = 82.3 ± 15.2) elite Para and able-bodied volleyball players from seven countries (Poland, Hungary, Serbia, Bosnia and Herzegovina, Slovenia, Italy, and France) took part in the study. The inclusion criteria were as follows: (1) Para or able-bodied male or female volleyball players, (2) at least two years of training experience at an elite level, (3) at least two sport-specific training sessions per week, (4) at least minimal disability (MD) according to the World Para Volley classification (only for Para athletes), (5) lateral lower limb amputation (only for Para athletes), (6) acquired impairment, and (7) satisfactory self-reported health status. The exclusion criteria were: (1) congenital impairment, (2) bilateral amputation or impairment different than lateral lower limb amputation (only for Para athletes), and (3) rejection from participating in the study. Participants were divided into three study groups i.e. study group 1 (SG1) of lateral amputee sitting volleyball players (female = 5; male = 26), study group 2 (SG2) of able-bodied sitting volleyball players (female = 4; male = 14), and study group 3 (SG3) of able-bodied Olympic volleyball players (female = 11; male = 14). The examinations that included SG1 and SG2 were carried out a day before the beginning of the Para Volley Euro League (Mysłowice, Poland), while an assessment of the players from SG3 was performed at the Jerzy Kukuczka Academy of Physical Education in Katowice, Poland a week before the beginning of the Polish League season. A detailed description of the study participants is presented in Table 1.

**Table 1:** The descriptive statistics and frequency tables of the study participants.

Variables	SG1 Lateral amputee sitting volleyball players (n = 31; nF = 5, nM = 26)	SG2 Able-bodied sitting volleyball players (n = 18; nF = 4; nM = 14)	SG3 Able-bodied Olympic volleyball players (n = 25; nF = 11; nM = 14)	Participants’ characteristics (n = 75; nF = 20, nM = 54)
	Mean ± SD	Mean ± SD	Mean ± SD	Mean ± SD
Age (years)	40.2 ± 9.1	38.2 ± 10.2	23.8 ± 5.04	34.1 ± 11.1
Body mass (kg)	84.9 ± 15.4	85.8 ± 12.7	76.6 ± 15.4	82.3 ± 15.2
Body height (m)	1.82 ± 0.1	1.81 ± 0.1	1.85 ± 0.1	1.83 ± 0.1
Sport-specific training experience (years)	10.7 ± 10.2	5.5 ± 5.37	12.0 ± 4.0	9.88 ± 7.8
Number of volleyball training sessions per week	2.6 ± 0.6	2.5 ± 0.6	7.0 ± 3.0	4.05 ± 2.8

n, Total number of participants; nF, Number of females; nM, Number of males; SD, Standard deviation.

The majority of amputee sitting volleyball players used prostheses (n = 27) while some of them used orthopedic crutches (n = 3) in everyday life. Moreover, the athletes from SG1 and SG2 did not have a specific playing position, while in SG3 the positions were as follows: setter (15%), opposite hitter (15%), outside hitter (19%), middle blocker (31%), and libero (15%). Moreover, athletes from each team were in the same training macrocycle. Study participants were allowed to withdraw from the experiment at any time and were informed about the benefits and potential risks of the study before providing their informed consent for participation. The research protocol was approved by the Bioethics Committee for Scientific Research at the Jerzy Kukuczka Academy of Physical Education in Katowice, Poland (No. 9/2012) and met the ethical standards of the Declaration of Helsinki, 2013.

### Procedures

All measurements were performed in the morning (7–11 a.m.) and started with individual interviews with study participants on their health status and sports history. Next, anthropometric measurements were performed: body height (using a stadiometer with a centimeter scale), and body mass (a chair weight). After the measurements, the prevalence and location of musculoskeletal complaints were assessed with the presence of an experienced researcher (EG) and according to the methodology of Kurionka et al.^15^ using a subjective Nordic Musculoskeletal Questionnaire for the previous seven days (NMQ-7) that is characterized by high validity (range of 80–100%) and reliability (range of 78–100%), and includes nine body parts: the neck, shoulders, upper back, elbows, wrists, lower back, hips/thighs, knees, and ankles/feet. Before completing the questionnaire, amputee athletes were instructed not to report phantom pain. The prevalence and locations of injuries and traumas were examined using a survey questionnaire by Zwierzchowska et al.^12^.

### Statistical analyses

All statistical analyses were performed using the statistical software IBM SPSS Statistics for Windows, version 21.0 (IBM Corp., Armonk, NY, USA). The quality data for analyzing variables were obtained using descriptive statistics and frequency tables (CrossTabs Commands). To classify the three study groups (lateral amputee sitting volleyball players, able-bodied sitting volleyball players, and able-bodied Olympic volleyball players) according to game-related statistics of injuries, traumas, and musculoskeletal complaints, a classification tree analysis based on the CRT algorithm was performed according to the methodology presented in the study by Gómez et al.^16^. The following specifications were assumed: (i) to avoid overfitting, a pruning procedure was applied in the CRT analysis, and the smallest tree with a cost within one standard error was chosen; (ii) the range of iterations was selected from 5 to 1 (maximum-minimum); (iii) the improvement measure could not be smaller than 0.001; (iv) the impurity measure was applied to maximize within-node homogeneity; (v) the missing independent values were excluded from the process and then surrogates were included in the descending tree; and (vi) to minimize the misclassification risk, a maximum five-level tree was selected and cross-validation was conducted. Each independent variable was ranked according to its importance to the model. Furthermore, the misclassification risks were calculated as a measure of the reliability of the model. The level of significance was set at *p* < 0.05.

## Results

The prevalence and location of musculoskeletal complaints based on the NMQ-7 questionnaire in all groups of studied athletes and SG1, SG2, and SG3 are presented in Table 2. The NMQ-7 questionnaire showed that in SG1, the musculoskeletal pain was located both in the upper and lower body parts, in which shoulders (36%), neck (29%), knees (26%), and upper and lower back (23%) were the most frequent painful areas. On the contrary, in SG2, only two body parts were indicated by the majority of players i.e., shoulders (44%) and knees (39%), while in SG3, low back pain (LBP)(52%) was reported as the most frequent location of musculoskeletal complaints, followed by pain in hips/thighs (36%) and shoulders (32%). Furthermore, both amputee sitting volleyball players and able-bodied Olympic volleyball players were characterized by an almost identical prevalence of reported musculoskeletal pain (60/62), while the lowest prevalence of pain was identified in able-bodied sitting volleyball players.

**Table 2:** The descriptive statistics, prevalence, location, and comparison of the musculoskeletal pain based on NMQ-7 in all groups of athletes: lateral amputee volleyball players (SG1), able-bodied sitting volleyball players (SG2), and able-bodied Olympic volleyball players (SG3).

Body parts (NMQ)	SG1 (n = 31) NMQ-7 number of athletes (n) and percentage (%)	SG2 (n = 18) NMQ-7 number of athletes (n) and percentage (%)	SG3 (n = 25) NMQ-7 number of athletes (n) and percentage (%)	All groups (n = 75) NMQ-7 number of athletes (n) and percentage (%)
Neck	9 (29%)	1 (5.6%)	3 (12%)	13 (17.6%)
Shoulders	11 (35.5%)	8 (44.4%)	8 (32%)	27 (36.5%)
Upper back	7 (22.6%)	1 (5.6%)	5 (20%)	13 (17.6%)
Elbows	6 (19.4%)	1 (5.6%)	4 (16%)	11 (14.9%)
Wrists	5 (16.1%)	1 (5.6%)	5 (20%)	11 (14.9%)
Low back	7 (22.6%)	2 (11.1%)	13 (52%)	22 (29.7%)
Hips/thighs	4 (12.9%)	0 (0%)	9 (36%)	13 (17.6%)
Knees	8 (25.8%)	7 (38.9%)	6 (24%)	24 (32.4%)
Ankles/feet	3 (9.7%)	3 (16.7%)	6 (24%)	12 (16.2%)
Sum of reported musculoskeletal complaints	60	24	62	146

Specific characteristics of the reported injuries are shown in Table 3. In most able-bodied sitting volleyball players, the last injury had occurred more than two years before the survey (56%), while players from SG1 and SG3 experienced injury mostly less than two years before the survey. Regardless of the location, the majority of reported injuries happened during training sessions and were acute injuries (SG1, SG3) or overload injuries (SG2). As a result, players from all studied groups frequently needed rehabilitation, especially in SG3, in which players were excluded mostly for 3 weeks from training.

**Table 3:** The descriptive statistics and prevalence of the characteristics of reported injuries in all groups of athletes: lateral amputee volleyball players (SG1), able-bodied sitting volleyball players (SG2), and able-bodied Olympic volleyball players (SG3).

Complaint reported	SG1 (n = 31) number of athletes (n) and percentage (%)	SG2 (n = 18) number of athletes (n) and percentage (%)	SG3 (n = 25) number of athletes (n) and percentage (%)	All groups (n = 75) number of athletes (n) and percentage (%)
An injury > 2 years	13 (41.9%)	3 (16.7%)	16 (64%)	32 (43.2%)
An injury < 2 years	10 (32.3%)	10 (55.6%)	9 (36%)	29 (39.2%)
Surgical treatment because of an injury	5 (16.1%)	5 (27.8%)	9 (36%)	19 (25.7%)
Rehabilitation because of an injury	15 (48.4%)	8 (44.4%)	19 (76%)	42 (56.8%)
No break in training because of an injury	11 (35.5%)	0 (0%)	3 (12%)	14 (18.9%)
1 week of break in training because of an injury	2 (6.5%)	3 (16.7%)	4 (16%)	9 (12.2%)
2 weeks of break in training because of an injury	0 (0%)	3 (16.7%)	7 (28%)	10 (13.5%)
3 weeks of break in training because of an injury	4 (12.9%)	3 (16.7%)	8 (32%)	15 (20.3%)
> 4 weeks of break in training because of an injury	0 (0%)	2 (11.1%)	0 (0%)	2 (2.7%)
< 4 weeks of break in training because of an injury	6 (19.4%)	2 (11.1%)	3 (12%)	11 (14.9%)
Injury during a training session	11 (35.5%)	7 (38.9%)	21 (84%)	39 (52.7%)
Injury during a competition	11 (35.5%)	6 (33.3%)	4 (16%)	21 (28.4%)
Acute injury	18 (58.1%)	7 (38.9%)	14 (56%)	40 (54.1%)
Overload injury	4 (12.9%)	8 (44.4%)	11 (44%)	22 (29.7%)

The location and comparison of reported injuries and traumas are shown in Table 4. Among amputee sitting volleyball players, the highest prevalence of injuries was found for the upper limb/s i.e., humeral joint (42%), fingers (39%), and elbow joint (26%). A similar tendency for humeral joint injuries was found in able-bodied sitting volleyball players (28%) and Olympic volleyball players (23%). However, contrary to Paralympic volleyball in Olympic volleyball players, injuries were mostly located in the lower limbs (knee joint, ankle). Surprisingly, knee joint injuries were frequently reported in all studied groups (see Table 4). Nevertheless, in general, amputee sitting volleyball players and Olympic volleyball players were characterized by an almost identical prevalence of injuries, which is similar to the reported prevalence of musculoskeletal complaints (see Table 2).

**Table 4:** The descriptive statistics and prevalence, location, and comparison of the reported injuries and traumas based on the survey questionnaire in all groups of athletes: lateral amputee volleyball players (SG1), able-bodied sitting volleyball players (SG2), and able-bodied Olympic volleyball players (SG3).

Location of injury	SG1 (n = 31) number of athletes (n) and percentage (%)	SG2 (n = 18) number of athletes (n) and percentage (%)	SG3 (n = 25) number of athletes (n) and percentage (%)	All groups (n = 75) number of athletes (n) and percentage (%)
Head	1 (3.2%)	1 (5.6%)	0 (0%)	2 (2.7%)
Neck	0 (0%)	3 (16.7%)	3 (12%)	6 (8.1%)
Humeral joint	13 (41.9%)	5 (27.8%)	6 (24%)	24 (32.4%)
Elbow joint	8 (25.8%)	1 (5.6%)	1 (4%)	10 (13.5%)
Hand	3 (9.7%)	3 (16.7%)	3 (12%)	9 (12.2%)
Fingers	12 (38.7%)	0 (0%)	4 (16%)	19 (25.7%)
Wrist joint	5 (16.1%)	2 (11.1%)	0 (0%)	7 (9.5%)
Upper back	0 (0%)	1 (5.6%)	0 (0%)	1 (1.4%)
Lower back	0 (0%)	2 (11.1%)	5 (20%)	7 (9.5%)
Spine	2 (6.5%)	0 (0%)	0 (0%)	2 (2.7%)
Chest	0 (0%)	0 (0%)	1 (4%)	1 (1.4%)
Stomach	0 (0%)	0 (0%)	1 (4%)	1 (1.4%)
Pelvis	2 (6.5%)	0 (0%)	0 (0%)	2 (2.7%)
Hip joint	1 (3.2%)	1 (5.6%)	0 (0%)	2 (2.7%)
Knee joint	7 (22.6%)	6 (33.3%)	11 (44%)	24 (32.4%)
Thigh	0 (0%)	1 (5.6%)	3 (12%)	4 (5.4%)
Ankle	0 (0%)	3 (16.7%)	13 (52%)	16 (21.6%)
Toes	1 (3.2%)	0 (0%)	1 (4%)	2 (2.7%)
Foot	4 (12.9%)	0 (0%)	1 (4%)	5 (6.8%)
Other	1 (3.2%)	0 (0%)	0 (0%)	1 (1.4%)
Sum of reported injuries	55	32	53	140
Types of traumas				
Scrape	7 (22.6%)	3 (16.7%)	6 (24%)	16 (21.6%)
Bruise	11 (35.5%)	7 (38.9%)	10 (40%)	28 (37.8%)
Sprain	4 (12.9%)	4 (22.2%)	10 (40%)	18 (24.3%)
Joint dislocation	2 (6.5%)	4 (22.2%)	6 (24%)	12 (16.2%)
Fracture	0 (0%)	2 (11.1%)	2 (8%)	4 (5.4%)
Soft tissue strain	3 (9.7%)	2 (11.1%)	4 (16%)	9 (12.2%)
Other	5 (16.1%)	1 (5.6%)	4 (16%)	10 (13.5%)
Sum of traumas	32	23	42	97

The prevalence of traumas was lower than that of injuries in all groups studied (see Table 4). However Olympic volleyball players indicated them more frequently than sitting volleyball players. The most frequent types of traumas were scrapes and bruises (SG1), bruises and sprains (SG2, SG3), and joint dislocations (SG2).

**Figure 1:**
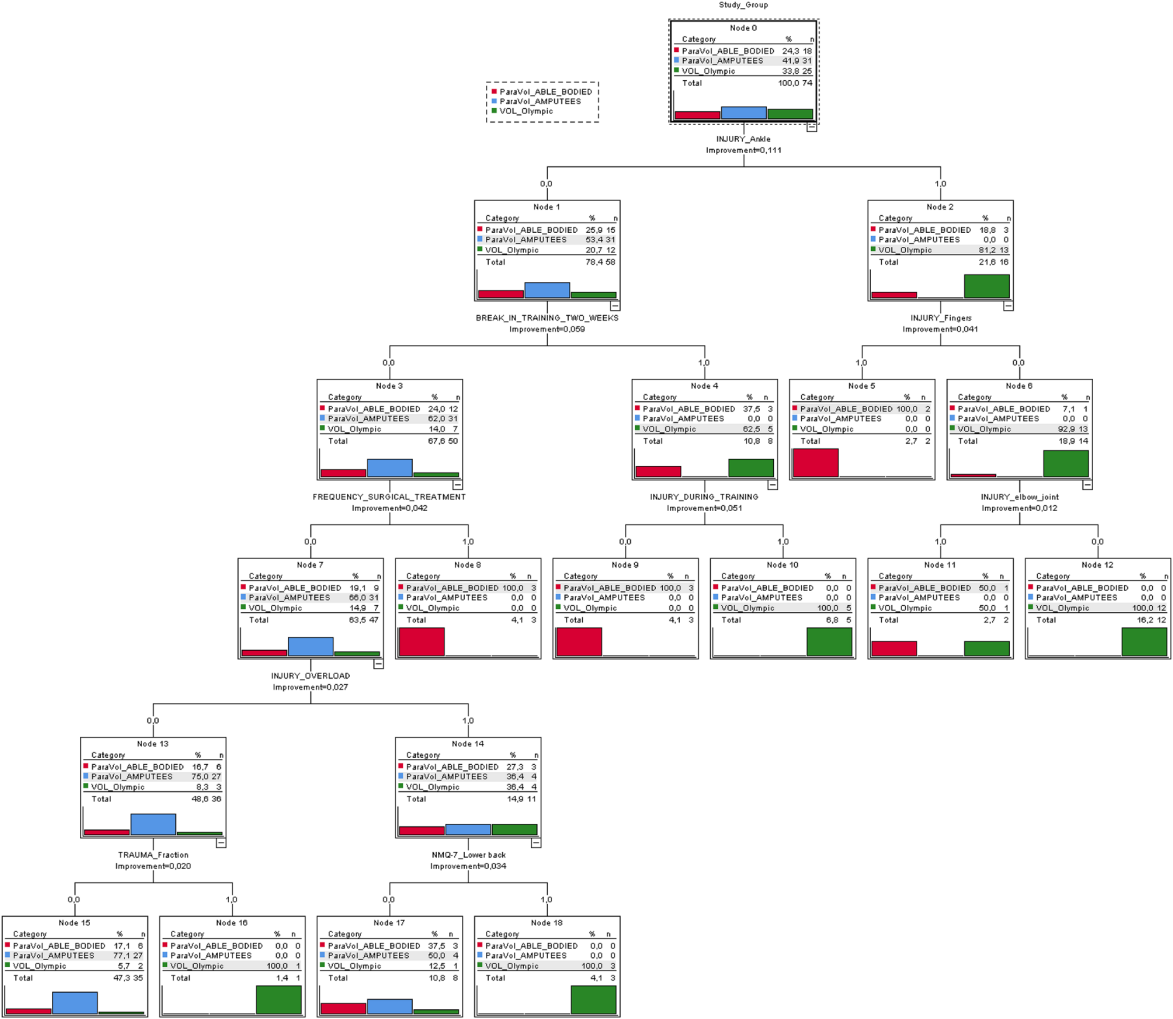
Categories for the predictor variable and 18 nodes—CRT analysis (created with: statistical software IBM SPSS Statistics for Windows, version 21.0—IBM Corp., Armonk, NY, USA).

Figure 1 presents the categories for the predictor variable and 18 nodes defined by the CRT analysis. For SG1, the CRT analysis showed seven significant factors on a five-stage tree, while in SG2 and SG3, nine significant factors were identified (Fig. 1). Eighteen nodes were mainly established by injury—ankle (level 1; improvement = 0.111), break in training for 2 weeks (level 2; improvement = 0.059), surgical treatment after injury, and injury during training sessions (level 3; improvement = 0.042 and 0.051, respectively), overload injuries (level 4; improvement = 0.027), fractures, and low back pain (level 5; improvement = 0.020 and 0.034, respectively) in both studied groups, while in SG2 and SG3, additional factors were identified, including injury—fingers (level 2; improvement = 0.041) and injury—elbow joint (level 3; improvement = 0.012).

## Discussion

The aim of this study was two-fold: (1) to identify the prevalence and location of injuries, traumas, and musculoskeletal complaints in Paralympic and Olympic volleyball players with different impairments and initial playing positions (sitting/standing); and (2) to identify the predictors of the abovementioned variables using a multivariate CRT model. The main finding of this study was that regardless of the impairment or initial playing position (sitting/standing), both the humeral joints and knee joints were found to be the most frequent locations of musculoskeletal pain and/or injuries in all studied groups, followed by low back pain that occurred in 2/3 of the studied groups, which partially supports our initial hypothesis.

Several reasons may explain the high prevalence of shoulder pain and humeral joint injury that was observed in different groups of athletes. Firstly, a systematic review performed by Challoumas et al.^17^ pointed out that Olympic volleyball training induces several morphological and biomechanical adaptations in the dominant shoulder that exhibit muscular imbalance. This results are in agreement with the studies by Shin et al.^18^ who indicated that decreased posterior tilt of the scapula along with glenohumeral horizontal abduction and dcapular internal rotations were associated with the incidence of shoulder pain and heighten risk of injury. Simultaneously the study by de Lira et al.^19^ indicated that lower level of shoulder internal rotational strenght can be associated with increased risk of shoulder pain and injury. Similar results were obtained in the study by Agel et al.^20^ who found that repeated spiking movement during volleyball is directly related to shoulder pain. It can therefore be assumed that in the volleyball players studied, the extrinsic compensatory mechanism was responsible for the high prevalence of shoulder pain and humeral joint injuries.

Secondly, the importance of the initial sitting position should be considered. Even though several systematic reviews enhanced that Olympic volleyball players are in general at the highest risk of overuse conditions in shoulders^17,21,22^, the current study found that both amputee and able-bodied sitting volleyball players had a greater prevalence of humeral joint pain and/or injury than able-bodied Olympic volleyball players. In addition, in amputee sitting volleyball players different musculoskeletal complaints and injuries are located mostly in the upper limbs i.e., neck, elbow joint, and fingers, which is in contrast to able-bodied Olympic volleyball players, in whom other types of musculoskeletal complaints and injuries occurred mostly in the lower body segments i.e., hips/thighs and ankle joint. Therefore, it seems that a forced sitting position during playing volleyball might induce a continuous overload of the shoulder girdle, greater than in Olympic volleyball, and may lead to both pain in the upper body’s segments and a higher risk of shoulder injuries. This thesis stands by agreement with the study by de Vries et al.^23^ who indicated that a increase in body height and in body mass can significantly heighten the risk of ankle and knee joints injury because of greater ovearload of the anatomical trails in the lower body parts during landing after jump, what is consistended with our results (able-bodied volleyball players). Simultaneously, based on the above, it can be postulated that because of forced sitting position in Paralympic volleyball, most of the biomechanical forces during moving and playing on the volleyball court are cumulated in the joints of the upper limbs, causing both acute/overuse injuries and muscular imbalance that may heighten the risk of injuries. Furthermore, lower limb impairment seems to additionally induce overload of the upper limbs as amputee sitting volleyball players were characterized by the highest prevalence of humeral joint injuries. Muscular imbalance of the rotational strenght profile, because of unilateral character of the spiking movement seems to be another issue that need to be addressed. The study by Ahmadi et al.^24^ indicated that sitting volleyball players tend to have asymmetrical rotational strength profiles, because of a constant overload of the upper body segments, which indicates the effect of compensatory mechanisms and is consistened with the results of our study.

Also interestingly, and somewhat surprisingly, knee pain and knee joint injuries were frequently reported in both groups of sitting volleyball players. It would seem that a sitting playing position during a volleyball game might contribute to isolating lower limbs and therefore decrease the risk of pain and/or injuries. Nevertheless, their overall prevalence was smaller than in Olympic volleyball players. Additionally, in Paralympic volleyball players, ankle injuries and significant types of traumas (e.g. sprains) were rarely reported. This can be easly explained by the studies of Bere et al.^10^ and Verhagen et al.^11^ who suggested that in case of Olympic volleyball players lower limb injuries (ankle and knee joints) are mostly resulted from contact with another player after landing. Thus it can be speculated that the initial sitting position may be a significant determinant of the prevalence of musculoskeletal pain, injuries, and traumas.

It should also be mentioned that both amputee Paralympic volleyball players and able-bodied Olympic volleyball players were characterized by an almost equal prevalence of injuries and musculoskeletal pain, which was not observed in able-bodied Paralympic volleyball players, what may be related to the initial playing position. A similar phenomenon was reported for the prevalence of low back pain (LBP). Based on the above, the sitting position seems to reduce the prevalence of musculoskeletal pain, injuries, and traumas, which explains the low prevalence of these variables in able-bodied Paralympic volleyball players. However, lower limb impairments can lead to a greater overload of the upper limbs, thus contributing to a higher prevalence of musculoskeletal complaints. The available research suggests that LBP is a typical complaint in Olympic volleyball, which may be related to decreased hip joint flexibility and shoulder flexors on the dominant upper limb^25^ due to extrinsic compensatory mechanisms. This is also consistened with the studies by Sponbeck et al.^26^ who found that volleyball training adaptations that occurs in the multifidus muscle cross-selection area are related to LBP in able-bodied volleyball players. What is more, our preliminary research suggested that forced sitting position in Para volleyball players may induce LBP because of disturbed pelvic tilt and lumbar concavity of the back^27^. Thus it should be highlighted that in Para sitting volleyball players, LBP could be related to both models of compensatory mechanism i.e., internal, as compensation and adaptation of the lower back and pelvic tilt to the acquired disability, and external, as an adaptation to disturbances in pelvic tilt while sitting during playing volleyball.

Furthermore, results of the present study showed the predictive importance of several variables (i.e., injuries in the ankle, fingers, and elbow joints, break in training for two weeks, surgical treatment, injury during training, overload injury, fractures, and low back pain) that were related to the prevalence of volleyball game-related effects on athlete’s functional status. In addition, the CRT analysis indicated the major effect of both lower limb amputation and initial playing position (sitting/standing) on the prediction of the abovementioned variables, which is consistent with our initial hypothesis. Based on CRT analysis (node 7, node 14) it could be concluded that the higher the volleyball training volume is, the greater risk of LBP occurs, especially in able-bodied Olympic volleyball players. Moreover, the prevalence of ankle injuries (node 0) seems to be the crucial predictor of further volleyball-related effects in different groups of volleyball players. These results provide valuable insight for athletes and trainers on sport-specific training volume that seems to play a significant role in musculoskeletal adaptations induced by extrinsic and intrinsic compensatory mechanisms.

## Limitations

The present study has several limitations that need to be addressed. First of all, we investigated three groups of elite Para and able-bodied Paralympic and Olympic volleyball players with various numbers of females in each group (SG1 = 5, SG2 = 4, SG3 = 14), which limits the generalization of the results. However, gender was not significant in statistical analyses for the verification of the study's aim and hypothesis. In addition, sitting volleyball players usually train and compete in different tournaments in mixed teams (males and females). Moreover, we found a similar tendency for the prevalence of musculoskeletal injuries and pain, which is the strength of the present study.

Secondly, there were age differences between Olympic volleyball players and Paralympic volleyball players. Nevertheless, it should be acknowledged that participants from SG1 had an acquired impairment (lateral lower limb amputation) that had caused permanent health damage. In most cases, the impairment had been a stimulus for participating in sport: firstly as a form of active rehabilitation and then as a form of self-realization and ability to compete at the highest level. Therefore, it is reasonable that sitting volleyball players were older than Olympic volleyball players.

The main strength of the present paper is the participation of a large group (n = 75) of elite-level Olympic and Paralympic volleyball players from different European countries. The sociodemographic diversity and numerous groups of study participants allow for inference concerning the impact of initial playing position (sitting/standing), volleyball training, and lower limb impairments on musculoskeletal pain, injuries, and traumas. Moreover, to author’s knowledge, this is the first investigation that focused on the identification of the predictors of musculoskeletal injuries and pain in Paralympic and Olympic volleyball.

## Conclusions

The current study can lead to the following conclusions: (i) firstly, extrinsic compensatory mechanism (initial playing position i.e., sitting/standing) may be a crucial variable for the prediction of musculoskeletal pain, injuries, and traumas in volleyball players; (ii) secondly, lower limb amputation seems to impact the prevalence of musculoskeletal complaints; and (iii) thirdly, training volume may predict the prevalence of LBP. Therefore, future studies should be extended to include more female players and anthropometric characteristics that may be relevant for the prediction of LBP. Such studies could provide important data that may enhance sports performance through the proper prevention of LBP by minimizing disturbances in body biomechanics.

## Data Availability

The datasets used and/or analysed during the current study available from the corresponding author on reasonable request.
